# Is a Hard (Or So-Called) Chancre a Sufficient and Reliable Evidence of Syphilis Infection?*Read before the Kansas City Academy of Medicine, Dec. 10, 1898.

**Published:** 1899-02

**Authors:** J. P. Knoche

**Affiliations:** Kansas City, Mo.


### IS A HARD (OR SO-CALLED) CHANCRE A SUFFICIENT AND RELIABLE EVIDENCE OF SYPHILIS INFECTION? *

BY J. P. KNOCHE, M.D. Kansas City, Mo.

It is not my purpose to enter into a lengthy argument as to the different opinions extant regarding the theories of unicity or quality of chancre virus. Among the advocates of both theories we find men of large experience and international reputation. Each of us can only speak with certainty of that which comes under our own experience. Beyond that we enter into the domain of the speculative and formulate our conclusions from inferences.

Dr. Edward B. Bronson, in his differential diagnosis of primary syphilis and simple chancre, advances the following points:

*Primary Syphilis.*—Often venereal. Produced by either mediate or immediate contagion; by inoculation not only of secretion from the initial lesion of syphilis, but from any exuding lesion of the secondary period, and possibly from certain genital discharges in syphilitic subjects not associated with syphilitic lesions.

*Simple Chancre.*—Almost always venereal. Almost always produced by immediate contagion, and only by inoculation of secretion from a chancrous lesion—*i. e.*, simple chancre or chancrous bubo.

*Incubation.*—Usually from a fortnight to a month.

*Incubation.*—Reaction within twenty-four hours.

*First Appearance.*—A pauple or slightly thickened eroded spot.

*First Appearance.*—A pustule or ulcer.

*Number of Lesions.*—Usually solitary; more rarely multiple, and then as a rule the multiple lesions appear simultaneous, seldom successively.

*Number of Lesions.*—Rarely solitary. Multiple lesions often develop successively.

*Seat.*—Not uncommonly extra-genital

*Seat.*—Almost exclusively on the genitals or their immediate vicinity.

*Induration.*—Is almost constantly present; is firm, elastic, and sharply defined.

*Induration.*—A rare accidental occurrence. When present, is a simple inflammatory thickening, that to the touch is compressible, inelastic, and ill-defined.

*Surface.*—Sometimes dry and scaling; more often moist, smooth; red or grayish.

*Surface.*—Always moist or incrusted, with grayish, uneven, pultaceous surface.

---

* Read before the Kansas City Academy of Medicine, Dec. 10, 1898.

4

*Form.*—Usually round or oval, with regular outline.

*Form.*—At first round, later angular in outline, with irregular borders.

*Ulceration.*—Not essential; often absent. Nearly always superficial; even when excavated or funnel-shaped, edges smooth and sloping.

*Ulceration.*—Essential. Deep sinuous, with perpendicular, undermined, or jagged edges.

*Secretion.*—Scanty and serous except under the influences of unusual irritation, when it may be abundant and purulent.

*Secretion.*—Copious, purulent; often sanious.

*Sensation.*--But slightly sensitive.

*Sensation.*—Sensitive.

*Inoculability.*—Inoculable upon a person not syphilitic.

Rarely auto-inoculable. Not inoculable upon a syphilitic person.

*Inoculability.*—Always inoculable upon others.

Auto-inoculable to an indefinite extent.

*Adenopathy.*—Polyadenitis; indolent; rarely suppurates.

*Adenopathy.*—Monadenitis; commonly acute, suppurating and virulent.

Bearing these points of distinction in mind it should be comparatively easy to distinguish the initial lesion of syphilis from a simple chancre, provided these lesions always preserve their typical character. But, unfortunately, this is not so. The microscope does not differentiate between chancre and chancroid. The cell infiltration of the derma and papillary proliferation and disintegration of the reta and the partial infiltration of the corium, hypertrophy of the adventitia of the blood vessels and contraction of the lumina will be found in both. Various syphilographers have at one time or another laid claim to the discovery of a germ specific of syphilis. All of such claims up to the present day have proven upon further investigation to be groundless in every case. An inoculation with a pure culture of the supposed syphilis germ has failed to produce syphilis. Lustgarten, in 1885, at that time an assistant in the clinic of Kaposi of Vienna, created quite a furor among syphiloligists by an announcement that at last a Moses had arisen to deliver the much-sought and hitherto evasive syphilis germ to the scrutiny of the medical profession. But this as many other like discoveries proved a fiasco, as inoculation with a pure culture of the discovered bacillus did not produce syphilis.

The diagnosis of chancre is not a matter of such extreme ease as we are at times led to believe; a superficial examination, a detection of induration and adenitis, and the rapid conclusion that we have a case of syphilis, may show at the time great diagnostic brilliancy, and later evidence a serious error of judgment. The existence of an induration is not, in my experience, an inflexible evidence of syphilitic chancre; although where syphilis is present it is safe to say that 92 per cent. of the chancres are indurated, and in 5 per cent. of the cases secondary eruption follow a sore where at no time could induration be discovered, upon a most careful and painstaking examination, and in about 3 per cent. of the cases no secondary symptoms follow a well-defined, indurated, primary sore. In several of the latter cases, after three to five years of observation, no evidence of syphilis have been found.

Polyadenitis is a constant accompaniment of syphilitic infection. When the chancre is located on the gland, penis or prepuce there will always be found enlargement of the lymphatic vessels on the dorsum of the penis. In those cases where the systemic conditions are especially favorable the lymphatic vessels enlarge to the size of a goose quill, inflammatory and painful, yet suppuration is rarely seen. Frequently the enlargement of the lymphatic vessels on the dorsum of the penis will be evident for some time before the lymphatic glands in the groin show enlargement.

No matter where the chancre may be located, we always find the lymphatic enlargement pronounced in the vicinity thereof. Because of this fact, I have several times traced an initial lesion, although in an unexpected and extraordinary location. The glandular enlargement, following an initial syphilitic lesion, presents certain peculiarities, especially upon palpation, conveying the sensation of a bag fully distended with liquid. To the pseudo-syphiloligist this latter condition has been the cause of many regrettable punctures to evacuate the pus that was not there. Adenitis due to syphilis only, is never inflammatory in the sense of a gonorrhœal or chancroidal bubo. It is claimed by some syphiloligists that they have found pus in bubos where all inflammatory indications, such as found in chancroidal bubos, were absent, and that because of the non-inoculability of the pus the conclusion was pure syphilitic adenitis. I have several times made this experiment with pus from a freshly opened chancreoidal bubo, and in neither case did I succeed in producing a chancroidal sore, although very different results followed where the secretion was taken from the initial lesion of chancroid.

A syphilitic chancre may be located anywhere on the human body, as the following cases, which have come under my personal observation, will show. Aside from the genital organs, the lips appear to be a favorite locality. In the examining of a suspicious sore on the lips, it is always well to bear in mind the possibility of epithelioma. This is especially to be observed in those cases that complain of frequently having a sore lip, due to fever blisters, and having enlarged cervical glands, which are spoken of as kernels. The history as given by the patient is often misleading, as I found in several cases treated as cancer and vice versa.

An indurated sore located on the glans penis, with enlargement of the lymphatics on the dorsum of the penis, and a painless inguinal adenitis does not always evidence syphilis, as the following case shows:

December, 1892, Mr. F. came to consult me regarding a sore on his penis with the following history and appearance: White, age 31 years, English parentage, strong and robust appearing, except for a well-marked cachexia.

No history of cancer or tubercular trouble in the family; had never had veneral disease; had frequently suffered with herpetic outbreaks on penis back of glands, which readily yielded to local measures, otherwise he had always enjoyed good health, until some time in the latter part of 1891 when in Canada he had intercourse with a woman, at a time before the total disappearance of one of these hepatic outbreaks; within twenty-four hours the unhealed hepatic spots became very inflamed and painful. This condition remained for several days. A physician diagnosed the case chancroid, and treated him for some time, and succeeded in curing all the places in a week or ten days, except one, on the corona glandis, near the frenum, which healed in thirty days. He then came to the United States; and when in New York several weeks later, this same place became sore again after sexual intercourse. From that time, for over a year, he had been treated by several physicians for syphilis, but the sore would not heal. When he came to me I found the following conditions:

An indurated sore as large as a nickel, located on the left back end of the glands, about one-third of the sore involving the glands penis. The sore had a scooped out appearance but somewhat ragged, undermined edges, the base a white and gray mottled, except a little patch on the upper side, which was red and granular; bled readily when swabbed with cotton; moderate thick pus secretion; marked induration and enlargement of the lymphatic vessel on the dorsum of the penis and the glands in the groin, especially on the left side; no pain or special discomfort, except when he had an erection; no sore throat, or loss of hair, or eruption at any time. I examined the patient thoroughly and could find no symptoms of syphilis except as heretofore set forth. I found no enlargement of the glands other than stated. Of the glands in the groin only two on the right side were moderately enlarged, while several on the left side were enlarged, one quite as large as a small hen's egg. I was in doubt as to the true nature of this trouble, so kept the patient under observation one week without treatment, at the end of which time I pronounced the sore epithelioma. I advised amputation of the penis and the desecting out the affected glands, which the patient refused to permit. After several weeks treatment the largest gland gave evidence of containing pus and was lanced and pus evacuated. The wound became very painful and then the patient consented to surgical interference. The penis was removed, also as many of the glands in each groin as could be reached. The patient died several days later. An autopsy revealed numerous cancer deposits in several vital organs (a pathologist upon microscopic examination pronounced the disease to be epithelial carcinoma).

It is always well to take time and make a careful examination, several in fact, as long as there is a lingering doubt about the correctness of the diagnosis. We often have nervous, fidgety women, who wish to hurry us, and if they are successful, we are usually wrong in our diagnosis. This occurred to me about eighteen months ago, when called in consultation. The woman had a suspicious sore in the vagina, just posterior and adjoining the meatus urinarius. Her physician had her on the table with speculum introduced and sore cleansed. I found an elongated and irregular sore about as large as a copper cent, inflamed and somewhat indurated, slightly tumified edges, gray mottled base, anterior half of sore bleeding. I wanted to examine the groin, mouth, etc , to which the patient objected. The doctor assured me that the glands in the groin were slightly enlarged, and the patient, that she had had a sore throat on numerous occasions. I without further examination, decided it was not a chancre, but a secondary syphilitic lesion. Later developments proved it cancer. This error could have been avoided, had a proper examination been insisted upon.

Chancre of the labia majora and minora are common, but not of the vagina. Out of many hundreds of cases I have seen but one. Even in this case it is a question if the initial lesion was not what is called a mixed chancre, involving the vagina from the osluteri. The roseloa existing at the same time with inguinal adenitis slightly more marked that the cervical and epitrochleor.

Regarding the unusal location of chancre, I have in my private practice observed the following: Once on the scrotum, twice on the monsveneris, once on the abdomen, near the umbili is, once on the cheek, once on the scalp and twice on the index finger.

This history of the case of chancre of thecheek is as follows: Mr. W., age 23, hearty, strong young man, when going home one evening met a friend who was decidedly under the influence of liquor. He forthwith took upon himself the good Samaritan act, took his friend home, and put him to bed. While undressing him this act of kindness so much impressed him that he threw his arms around W. and playfully bit him on the left cheek. Sixteen days thereafter W. came to me with five distinct little indurated chancres, two evidencing the outline of the incisors and three the lower teeth. The two indurations above and the three below were joined with a space of three-fourths of an inch between them of unaffected tissue. The submaxilliary glands especially on the left side were hugely enlarged. Secondary manifestations followed in due time.

The chancre on the scalp was located on the center and top of the head. The patient was a short man medium built. He came to me regarding an eruption on his body, and an examination proved it to be maculopapular sphilide. He absolutely denied he had ever had a sore of any kind. I had him strip, and examined him thoroughly. The cervical and both post occipatal glands were most enlarged. I at last found a healed, but yet indurated scar not more than one-fourth inch long and narrow, on the top of his head. When I asked him about it he laughingly told me that some three or four months before he was copulating with a very tall woman, and being a very short man, his head only reached to her breast, and then during that period, when faith, hope and charity are blended in one, she bit him on the head. Though he said it did not hurt him at any time except for a few days and then when brushing his hair.

The case of chancre of the abdomen was brought about by the patient manipulating the vagina and vicinity thereof, of his female companion, prior to intercourse. The female (as later examination proved) had syphilitic papules around the edge of the vulva. The patient in his manipulations propably got some of the virus under his finger-nail, and as he suffered from prickly heat, as he claimed, he undoubtedly in scratching denuded some pimple at the point of inoculation, thereby receiving the poison into his system. The proof that this was the initial lesion was evidenced in due time by secondary manifestation.

The following cases will be of interest as evidencing the difficulties ofttimes encountered by the diagnostician:

Mr. C., postal railway clerk, family history good, age of patient 27, health good, never had venereal disease, for several years suffered from herpes preputialis; the attacks of this latter trouble did not persist longer than a week or ten days and always yielded promptly to treatment. He came to me in the latter part of 1888 with the following history: About five months before, on a Saturday night, had intercourse with a woman; observing the usual precaution of washing, he noticed no bad effect until the following Monday night. He was at this time in a mail car, working an especially heavy mail, between Kansas City and St. Louis. The trouble began as scalding urine, followed by burning and itching of the part underlying the prepuce. This condition continued more or less all night. By Tuesday morning the prepuce was very odematous and the affected parts burning and painful. He had also fever, headache and vomiting. He was too sick to take his run out, but came to Kansas City and then went to an adjoining city to consult a medical friend, who after several days' treatment reduced the odema sufficient to retract the prepuce and expose the affected part. Here he found an excoriated surface supporting three well-defined ulcers, distinctly indurated. Enlargement of the lymphatics well marked on both sides. Within seventeen days from the period of exposure, the patient had an eruption break out on him, which disappeared in one week. The diagnosis made by the physician was syphilis, and the patient put on mercurial treatment. When he came to me two months later I readily found the scars at the former seat of the ulcers, but no induration of the lymphatic vessel on the dorsum of the penis. The lymphatics in the groin and neck could be plainly felt as enlarged. The glands were flattened and had a sclerotic feel; even the epitrochlear glands were slightly enlarged. None of the glands had the peculiar feel found in syphilis. There had been at no time sore throat, loss of hair or other syphilitic symptoms, but this might be accounted for on the ground of treatment. After careful and repeated examinations, and finding a lack of specific evidence, and the history of the case and the patient's occupation, I decided against syphilis. The patient has been under observation for ten years, and up to the present no symptoms of syphilis have appeared. He is also the father of several robust children. I have made numerous examinations of railway postal clerks, and in every instance I have found enlargement of the inguinal, in 90 per cent. of the cervical, and in about 60 per cent., of the epitrochlear glands, and this applies only where there was no history or evidence of syphilis. The constant tension on the extremities, to maintain equilibrium in a fast-moving, swaying car, the movement of the head in locating the receptacles for the distributing of mail, fully accounts for the enlarged lymphatic glands. It is therefore well to learn the occupation of your patient, as it may furnish an additional guide for a correct diagnosis.

It is well to bear in mind that in all cases where we have a history of recurrent herpes preputialis, and the patient has had syphilis, although a number of years have passed since the initial lesion, we may have at any time a well-defined sclerotic induration at one or more (usually one, rarely more than two) places just posterior to the glands; this is especially true if upon the circumscribed area the irritation has persisted more or less constantly for several weeks. Frequently we find that the patient has succeeded in conquoring the herpes, and the existing and non-painful nodule drives the patient to seek the advice of a physician. I have had several of these cases within the last year. The last case, only ten days since, showed a well-defined circumscribed induration as large as a split pea, located just back of the glands and midway between the frenum and dorsum of penis. The surface was slightly denuded but not ulcerous. There was no enlargement of the lymphatic vessel on the dorsum of the penis, the glands in the groin were slightly larger than normal, the patient had syphilis ten years before, had taken a year's treatment and course at Hot Springs.

The absence of lymphatic enlargement can, I believe, be accepted with a degree of safety, as against ths existence of a primary initial lesion.

Where a patient has been cured of syphilis and becomes re-inoculated, the glandular enlargement is as pronounced, and the secondary eruptions following the same, as though he had not had the disease before. I am aware that much discredit is cast upon all writers claiming to have treated cases of second infection of syphilis. I am fortunate to have seen three cases of this kind, one in the clinic at Vienna and two in my private practice. In each of the latter cases I saw the primary sore, the accompanying glandular enlargement, and secondary eruption. Each case was under my care and treatment for two years. Number one, five years and three months after the period of first inoculation, came to me again with an undurated sore, glandular enlargement, as in the first instance, and in due time a maculo-papular syphilide. Number two was re-inoculated four years and one month after the period of first exposure. In this case as in number one the same decided glandular enlargement, the secondary eruption manifesting as a roseola, eight weeks after the appearance of the chancre. In neither case at the first treatment did I observe a symptom of syphilis after ten months. Both of these patients are now under treatment.

Various methods of treating chancre have been suggested to eradicate or modify the constitutional effect of syphilis. It is claimed by various writers, that excision of the chancre has ofttimes proved successful in radically modifying or aborting further syphilitic manifestations. But in a large majority of cases this method of treatment has utterly failed of effect on the secondary manifestation. In my practice I have resorted four times to this method of treatment. In each case the chancre was typical and well defined and located on the prepuce, thereby permitting thorough removal. In each case the lymphatic vessel on the dorsum of the penis and inguinal adenitis was well marked. I was fortunate enough to see these cases within the first week of the appearance of the sore. Two of the cases here referred to had violent manifestations of secondary syphilis. Number one a papulo-squamous and and the other a papulo-pustular syphilide. The other two evidenced the usual roseola. In one of the latter cases I encountered a most persistent recurrent specific ulceration of the tonsils. Equally useless have I found injections of corrosive sublimate solution into and around the induration. A slough and greater sore was the only result. The mild chloride and yellow oxide are equally useless as abortives or modifiers. The early inunction before the secondary eruption (where syphilis is present) only succeed in delaying them. I have seen roseola appear thirty days after the cessation of a three-months' course of inunction. The only difference being that the roseola spots are less in number and of larger size.

That syphilis is caused by a specific microbe I firmly believe, although I can not prove it. No one so far has succeeded in finding a germ, a pure culture of which would produce syphilis. Thousands of attempts have been made to inoculate various animals, all to no purpose. It is only in the body of man, let him be of high or low degree, that syphilis finds a fertile field. We have no adequate proof of its presence in the body until evidenced by a secondary manifestation.

Accepting the precaution that experience has taught, namely, the uncertainty accompanying the diagnosis of syphilitic chancre, based alone upon the sclerotic induration of a sore and glandular enlargement, I am constrained to the opinion that in all cases of excision, where the secondary syphilitic manifestation remain absent, the failure of such secondary manifestations to appear, is not conclusive proof that the excised sore was due to syphilitic infection. My experience does not permit me to assert that we can with safety determine the absence, or presence, of syphilis from the initial lesion, no matter if circumscribly indurated, and an accompanying adenitis; although such induration and adenitis furnish strong presumptive evidence, conclusive proof is shown only by the secondary syphilitic manifestation. In conclusion I would advocate the treating of all suspicious initial lesions with some simple dressing such as nosophen, boracetanilid, aristol or bismuth subgallate, and I wish to reiterate that under no circumstances are we justified to reckon the patient as a syphilitic on the evidence of an indurated sore and accompanying glandular enlargement, and subject him to a long course of treatment and the accompanying influence on his future career, unless we have the qualifying evidence of the secondary manifestation.—*Kansas City Lancet.*

